# Center of Pressure Motion After Calf Vibration Is More Random in Fallers Than Non-fallers: Prospective Study of Older Individuals

**DOI:** 10.3389/fphys.2018.00273

**Published:** 2018-03-26

**Authors:** Wolbert van den Hoorn, Graham K. Kerr, Jaap H. van Dieën, Paul W. Hodges

**Affiliations:** ^1^Centre for Clinical Research Excellence in Spinal Pain, Injury and Health, School of Health & Rehabilitation Sciences, The University of Queensland, Brisbane, QLD, Australia; ^2^Movement Neuroscience Program, Institute of Health and Biomechanical Innovation, Queensland University of Technology, Brisbane, QLD, Australia; ^3^Amsterdam Movement Sciences, Department of Human Movement Sciences, Vrije Universiteit Amsterdam, Amsterdam, Netherlands

**Keywords:** aging, muscle vibration, balance, somatosensory, proprioception, falls, recurrence quantification analysis, detrended fluctuation analysis

## Abstract

Aging is associated with changes in balance control and elderly take longer to adapt to changing sensory conditions, which may increase falls risk. Low amplitude calf muscle vibration stimulates local sensory afferents/receptors and affects sense of upright when applied in stance. It has been used to assess the extent the nervous system relies on calf muscle somatosensory information and to rapidly change/perturb part of the somatosensory information causing balance unsteadiness by addition and removal of the vibratory stimulus. This study assessed the effect of addition and removal of calf vibration on balance control (in the absence of vision) in elderly individuals (>65 years, *n* = 99) who did (*n* = 41) or did not prospectively report falls (*n* = 58), and in a group of young individuals (18–25 years, *n* = 23). Participants stood barefoot and blindfolded on a force plate for 135 s. Vibrators (60 Hz, 1 mm) attached bilaterally over the triceps surae muscles were activated twice for 15 s; after 15 and 75 s (45 s for recovery). Balance measures were applied in a windowed (15 s epoch) manner to compare center-of-pressure (CoP) motion before, during and after removal of calf vibration between groups. In each epoch, CoP motion was quantified using linear measures, and non-linear measures to assess temporal structure of CoP motion [using recurrence quantification analysis (RQA) and detrended fluctuation analysis]. Mean CoP displacement during and after vibration did not differ between groups, which suggests that calf proprioception and/or weighting assigned by the nervous system to calf proprioception was similar for the young and both groups of older individuals. Overall, compared to the elderly, CoP motion of young was more predictable and persistent. Balance measures were not different between fallers and non-fallers before and during vibration. However, non-linear aspects of CoP motion of fallers and non-fallers differed after removal of vibration, when dynamic re-weighting is required. During this period fallers exhibited more random CoP motion, which could result from a reduced ability to control balance and/or a reduced ability to dynamically reweight proprioceptive information. These results show that non-linear measures of balance provide evidence for deficits in balance control in people who go on to fall in the following 12 months.

## Introduction

Falls and falls related injuries are a serious health issue (Hill et al., 2004) in the aging population and poor balance control is a major contributor (Campbell et al., 1989; Maki et al., 1994). Balance control requires sense of the body's vertical with respect to gravity and sense of deviations away from the vertical with the goal to maintain the body's center of mass within the base of support (Horak, 2006). In addition to overall perception of orientation with respect to gravity, mostly provided by the vestibular system (Day and Fitzpatrick, 2005), feedback of the relative positions and movements of body segments is provided by somatosensation, and global orientation and movement is provided by vision (Proske and Gandevia, 2012). Sensory information is dynamically processed by the central nervous system (CNS), and appropriate corrections are applied by the motor system. Physiological aging is associated with diminished functioning of these systems and underpins some of the decline in balance control (Lord et al., 1991). Why some older individuals fall whereas others do not might plausibly be explained by variation in the decline of the somatosensory input and the impact of somatosensory changes on balance control.

Somatosensory information from muscle spindles in postural muscles is important for standing balance control (Horak, 2006; Proske and Gandevia, 2012). Somatosensory function can be assessed with low amplitude vibration of the muscle-tendon complex, which increases the discharge rate of muscle spindle Ia afferents (Burke et al., 1976; Roll et al., 1989) in a 1:1 relation with the vibratory stimulus (Roll et al., 1989), and creates an illusion of muscle lengthening (Goodwin et al., 1972). If the vibrated muscle serves a postural function, the illusory change in muscle length induces an illusory change in the sense of upright, and posture is automatically adjusted (Eklund, 1972; Barbieri et al., 2008). The magnitude of corrective center-of-pressure (CoP) displacement (i.e., reflection of the postural adaptation) reflects both the *sensitivity* of muscle spindles to vibration and the *relative weighting* that the CNS places on the contribution of the spindle input to the perception and control of posture (Brumagne et al., 2004).

The CoP response to triceps surae (calf) vibration in standing is affected by age, but findings are inconsistent. Postural responses of older individuals have been reported to be less (Pyykkö et al., 1990; Quoniam et al., 1995; Hay et al., 1996), more (Maitre et al., 2013), or similar (Brumagne et al., 2004; Abrahamová et al., 2009) to those in young individuals. Although, this variation in outcomes can partly be explained by differences in participant ages (Brumagne et al., 2004), differences in postural perturbation paradigms and small sample sizes, variable findings could also suggest that age-related changes in somatosensory functioning vary between individuals, placing some individuals at higher risk for falling.

Changes in the environment in daily life (e.g., lighting and support surface conditions) require constant re-weighting of somatosensory information to aid balance control. Aging affects the ability to flexibly reconfigure proprioception for postural control to changes in proprioceptive context (Hay et al., 1996; Sturnieks et al., 2008; Eikema et al., 2013, 2014). Sense of upright and balance control are perturbed by both addition and removal of the muscle vibration stimulus. Addition of vibration distorts part of, and contradicts, the total afferent source, causing balance unsteadiness (Eklund, 1972). Removal of vibration can cause the illusory change in upright posture to reverse (Wierzbicka et al., 1998; Duclos et al., 2007) again inducing balance unsteadiness. Balance unsteadiness after vibration removal is likely to be mediated, at least in part, by a transient reduction of discharge/sensitivity of muscle spindles (Rogers et al., 1985), and by time required by the CNS to dynamically re-weight available sensory systems (Brumagne et al., 2004; van der Kooij and Peterka, 2011). The ability to flexibly explore somatosensory redundancy (i.e., *re-weighting*) could be beneficial for balance control to minimize the perturbation effects on balance caused by addition and removal of muscle vibration. If not, this might result in increased unsteadiness during and after removal of the vibratory stimulus which could be linked with falls risk.

Linear measures of balance parameters such as sway path length or root mean square (RMS) velocity implicitly assume that the temporal structure of CoP motion arises from random fluctuations in the postural control system that do not change over time. These measures have been used in most investigations of the effect of muscle vibration on postural control. Although linear measures are affected by vibration, they offer little insight into the dynamic characteristics of CoP motion in response to vibration perturbations, which is likely to aid interpretation of the underlying mechanisms. Non-linear measures such as recurrence quantification analysis (RQA) (Eckmann et al., 1987; Marwan et al., 2007) and detrended fluctuation analysis (DFA) (Peng et al., 1995b) describe the temporal structure of CoP motion. The adaptable multisensory integration and response generation of optimal balance control (Nashner, 1976) results in balance performance that is resilient to small perturbations; quantified using RQA as a measure of the structure of recurrent CoP motion (Riley et al., 1999; Marwan et al., 2007), appears smooth and persistent; which is measured with DFA (Peng et al., 1995b). Measures obtained with these non-linear methods change when postural control is challenged (Riley et al., 1999; Riley and Clark, 2003), and can distinguish elderly from young individuals (Norris et al., 2005; Amoud et al., 2007; Duarte and Sternad, 2008; Kim et al., 2008; Seigle et al., 2009), although findings vary, (Seigle et al., 2009; Wang and Yang, 2012). These non-linear measures are likely to provide a more detailed understanding of how sensory perturbations impact balance control.

This study aimed to: (i) compare CoP motion between young and older individuals before, during and after removal of bilateral calf vibration, and (ii) compare measures between older individual who subsequently do or do not go on to fall in the following 12 months. We probed this question using linear and non-linear measures of CoP motion to investigate impact of addition and removal of vibration to the calf muscles.

## Methods

### Participants

One-hundred-and-six participants older than 65 years of age volunteered for this study (42 female, 64 male) with a mean ± *SD* age, weight and height of 75 ± 6 years, 78 ± 15 kg, 1.69 ± 0.09 m, respectively. Participants were a subset from a larger cohort (*n* = 252), and were included in the current study based on the Physiological Profile Assessment score (PPA, short form version) of Lord et al. (2003). To ensure a wide range of falls risk, participants were included in the current study if their PPA values were below 0.5 (*n* = 59) or above 1 (*n* = 47). All participants were recruited from the Brisbane metropolitan area via the Australian electoral role. A letter of invitation was sent with an information sheet, which outlined the potential risks and benefits of the research. Participants were excluded if they had a recent or recurrent history of surgery or musculoskeletal injury, any neurological impairment such as Parkinson's disease, were unable to ambulate independently without the use of a walking aid, or were cognitively impaired (i.e., Mini mental state exam score <24). The young group included 23 participants between 18 and 25 years of age (14 female, 9 male, 21 ± 2 years, 65 ± 10 kg, 1.72 ± 0.05 m) recruited from the student population of local universities and by word of mouth. All participants provided written informed consent. The experimental protocol was approved by the Institutional Human Research Ethics Committees and conformed to the Declaration of Helsinki.

### Prospective falls measurement

Elderly participants were followed for 12 months after the balance assessment. They maintained a weekly falls diary, which they returned at the end of each month via reply paid post (Hannan et al., 2010). A fall was defined as: “an unintentionally coming to the ground or some other lower level, not as a result of a major intrinsic event (e.g., stroke) or overwhelming hazard. This included any slips, trips or accidents, which result in a fall onto a lower level, be it a chair, bed or the floor for example.” A “faller” was defined as a person who had one or more falls recorded within the 12-month follow-up period.

### Experimental setup and procedure

Participants stood barefoot on a force plate (Type 9286AA, Kistler Group, Winterthur, Switzerland), were blindfolded to exclude the contribution of vision to balance, and wore headphones playing white noise to limit distraction. Participants stood relaxed with arms hanging by their sides and data collection commenced after ~20 s to ensure balance had reached a steady state.

Participants stood for 135 s after commencement of data recording. Custom-made vibrators (Type YM2707, Electus Distribution, Sydney, Australia, ~1 mm, 60 Hz) were bilaterally attached halfway between the distal portion of the gastrocnemius muscle heads and the distal insertion of the Achilles tendon. Firm application was assured with pressure applied to the vibrators using a neoprene band wrapped around the ankle and vibrator. After 15 s, mechanical vibration was applied bilaterally for 15 s. Vibrators were switched on for a further 15 s period at 75 s from commencement of recording. This allowed 45 s after cessation of each vibration exposure to assess post-vibration effects on balance. The experimenter stood close to the participant to provide support in case of falling. If balance was assisted, data collection was stopped and restarted if the participant agreed.

Force plate data were amplified [Type 5233A, (range: Fx & Fy; 250N, Fz; 2500N), Kistler Group, Winterthur, Switzerland] and digitized with 16-bit precision at a sampling rate of 2,000 samples/s using a Power 1401 data acquisition system with Spike2 software (Cambridge Electronic Design Limited, Cambridge, UK).

### Data analysis

**Figure 1:**
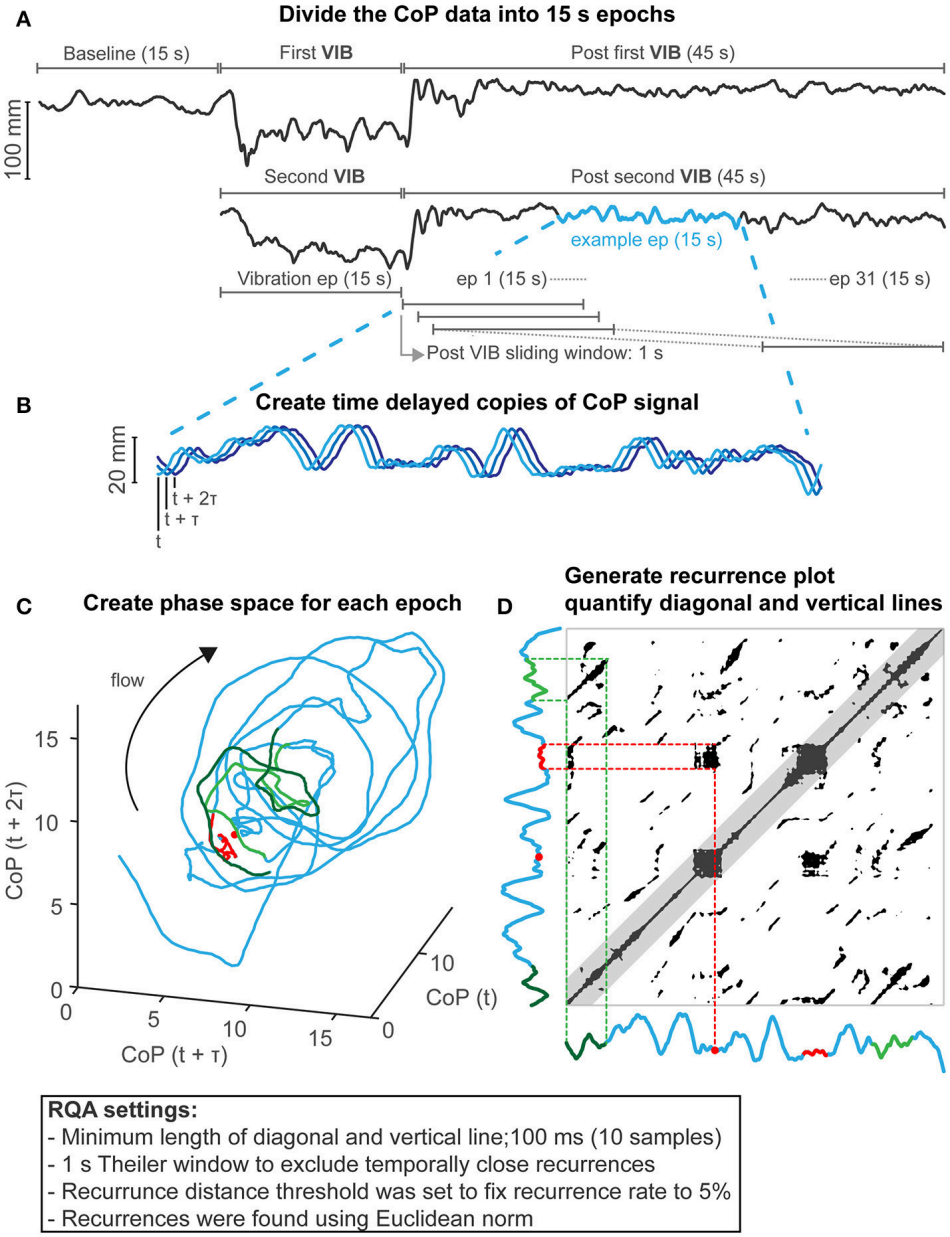
Recurrence quantification analysis methods. **(A)** CoP motion in anterior-posterior example of a participant (faller) showing baseline, first and second vibrations (VIB) and 45 s after each vibration. Data were analyzed using 15-s epochs (ep). This includes the vibration epoch, and epochs 1–31 (post vibration epochs), which started after cessation of vibration and was shifted in time with 1-s intervals (93.33% overlap) until 45 s after vibration to assess balance after vibration. This resulted in two sets (two vibration repetitions) of 32 epochs (1 vibration + 31 post vibration) for each participant which were used for statistical analysis to assess group differences. Group differences at the baseline epoch were assessed separately as there was only 1 repetition available (see Statistics section for more details). **(B)** Example of a CoP epoch (blue) delayed with a tau of 180 ms. **(C)** A phase space was created by plotting the delayed CoP copies against each other. Note that the example is given in 3D, but, analysis was performed in 5D. **(D)** The recurrence plot represents the recurrences of CoP in the phase space depicted in **(C)**; by creating a 2D recurrence plot by adapting the recurrence threshold distance to fix the recurrence rate to 5%. Temporally close recurrences were excluded (<1 s, Theiler window) which is represented by the grayed area along the line of identity (were CoP recurs with itself). Two examples are shown that represent a diagonal (in light and dark green) and vertical recurrence structures (in red). These examples are also shown in the phase space in **(C)**. The light and dark green represents CoP motion running parallel in phase space and the red line represents CoP motion that revisits and remains in a region in phase space represented by the red dot in **(C)** and **(D)**.

Data were analyzed offline with Matlab (Mathworks inc., Natick, MA, USA). As calf vibration mainly perturbs balance in the anterior-posterior direction (Eklund, 1972), analyses were focused on CoP motion in this plane. CoP data were filtered using a second order low pass bi-directional Butterworth filter. Cut-off frequency was set at 20 Hz and bi-directional filtering increased the filter order to 4. After low-pass filtering, data were decimated to 100 samples/s. All measures were applied in a windowed (15-s epoch) manner (Riley et al., 1999; Webber and Marwan, 2014), to assess changes in CoP motion during and after calf vibration (see Figure 1 for details).

#### Description of CoP motion

The CoP is the baricenter of the contact surface of an individual on the ground, or in other words the point of application of the ground reaction force. CoP motion provides a proxy measure of standing balance dynamics. CoP motion contains information regarding the motion of the vertical projection of the center of mass of the whole body and of the moments that are generated by the individual (Winter, 1995). For example, forward body lean involves a forward position of the center of mass, which is reflected in a forward position of the CoP. Moving the center of mass backwards by rotation around the ankle joints requires generation of ankle moments which shift the CoP further anterior relative to the center of mass.

#### Linear measures

Sway path (SP, mms^−1^) was calculated as the sum of the absolute distances between consecutive data points divided by epoch length. CoP position (mm), relative to the mean CoP position at the baseline pre-vibration epoch, was calculated as the mean position of CoP within each epoch.

#### Non-linear measures

##### Recurrence quantification analysis

CoP dynamics were captured by plotting time delayed copies of the CoP signal against each other [methods of delay (Takens, 1981), see Figure 1C for a 3-dimensional representation]. The phase space dimension was fixed at 5 dimensions determined with false nearest neighbor analysis (Kennel et al., 1992) using the whole signal (135 s). The delay was calculated using the average displacement method (Rosenstein et al., 1994) also using the whole signal, for each participant individually. Phase space dimension was limited to 5 dimensions as higher dimensional phase space would require longer time series (Grassberger and Procaccia, 1983; Marwan, 2011) and true recurrences might be missed (Marwan, 2011). The points in this volume (or phase space), represent the history of all balance solutions (or states). Recurrences of balance solutions within this phase space were visualized by a 2-dimensional recurrence plot (Eckmann et al., 1987), which represents the times at which balance solutions revisit (recur) in phase space (Webber and Marwan, 2014). RQA describes the features of these recurrences. Figure 1 shows the details of the RQA method and settings used. Table 1 provides definitions and interpretations of RQA parameters in relation to CoP motion.

**Table 1:** Definition and interpretation of recurrence quantification analysis (RQA) and detrended fluctuation analysis (DFA) in the context of the balance task.

	**Variable**	**Definition**	**Higher value interpretation**	**Lower value interpretation**
RQA diagonal	%DET	The percentage of all recurrences in phase space (below a pre-set threshold distance) that form diagonal line lengths longer than 100 ms	More predictable, more deterministic, less random CoP motion, consistent with better balance performance	Less predictable, less deterministic, more random CoP motion, consistent with reduced balance performance
	L_mean_	The mean length of the diagonal lines in the recurrence plot	Better balance performance, less impact of small perturbations resulting in more similar temporal dynamic CoP patterns	Reduced balance performance, greater impact of small perturbations, resulting in less similar (more random) temporal dynamic CoP patterns
RQA vertical	%LAM	The percentage of all recurrences in phase space (below a pre-set threshold distance) that form vertical line lengths longer than 100 ms	More intermittent CoP motion with more periods of minimal CoP fluctuations	Less intermittent CoP motion with fewer periods of minimal CoP fluctuations
	TT	The mean length of the vertical lines in the recurrence plot	Longer periods of minimal CoP fluctuations (static states)	Shorter periods of minimal CoP fluctuations (static states)
DFA	DFA_1_	Exponential interrelation of CoP fluctuations at time scales between 0.1 and tau s	Smoother and more persistent CoP motion at time scales < tau	Less smooth and less persistent CoP movements at time scales < tau
	DFA_2_	Exponential interrelation of CoP fluctuations at time scales between tau and 4.42 s	Smoother and less anti persistent CoP motion at time scales > tau	Less smooth and more anti persistent CoP motion at time scales > tau
	DFA_tau_	The time scale that separates DFA short and DFA long	Less conservative balance control	More conservative balance control

Recurrence quantification analysis (RQA), diagonal line structures; percentage determinism (%DET), mean diagonal line length (L_mean_), vertical line structure; percentage laminarity (%LAM), mean vertical line length (trapping time, TT). Detrended fluctuation analysis (DFA), short term DFA (DFA_1_), longer term DFA (DFA_2_) and the time scale that separates DFA_1_ and DFA_2_; DFA_tau._

The features of the recurrence plot using RQA were quantified by the diagonal lines; the percentage determinism (%DET), mean diagonal line lengths (L_mean_), and vertical lines; the percentage laminarity (%LAM) and trapping time (TT) using Marwan's RQA toolbox (Marwan, 1998; Marwan et al., 2007). To avoid ceiling effect of the %DET and %LAM, sensitivity was reduced by considering 0.1 s as a minimal length of both diagonal and vertical line features (Seigle et al., 2009; Ramdani et al., 2013).

The level of recurrence rate impacts the recurrence quantification (Riley et al., 1999). Therefore, the recurrence threshold, below which a recurrence was defined, is usually dependent on some measure of CoP motion amplitude, such as percentage of the maximum diameter of balance states within the phase space (Ramdani et al., 2013; Decker et al., 2015) or percentage of mean distance between al data points in phase space (Riley et al., 1999; Riley and Clark, 2003). However, because the size of the diameter of balance states is biased by larger CoP motion excursion and because not all areas in phase space will be revisited equally frequently, the amplitude measures that are used to set the recurrence threshold could skew the resulting recurrence rate, and therefore the recurrence quantification. This would be more likely to be problematic in shorter time series. Therefore, we adapted the recurrence threshold to fix the recurrence rate to 5% to avoid these issues and to have a more scale free RQA and to enable better comparison between groups at each CoP window.

*Diagonal line features*. Diagonal line features extracted from the recurrence plot, reflect the deterministic behavior of CoP motion, respectively (Figures 1C,D). The percentage of recurrences that form diagonal lines (%DET) and the mean diagonal line length (L_mean_) are positively linked with the predictability, i.e., the deterministic pattern of CoP motion, as similar balance solutions (states) will lead to similar CoP temporal patterns (Webber and Marwan, 2014). Diagonal structures are also linked with a real-life notion of stability (Marwan, 2011; Webber and Marwan, 2014). Consider two points in phase space that start as close neighbors and are followed over time. The length of the diagonal line represents the time that these points remain close (Figures 1C,D, dark and light green CoP motion example). The initial distance between the neighboring points at the start could be viewed as a small perturbation, i.e., a small difference in initial conditions, and the length of the diagonal line reflects whether balance control is affected by these small perturbations. Longer diagonal lines indicate balance control that is minimally affected by these small perturbations. Balance control must deal with these small perturbations to remain stable as upright stance can be viewed as an inverted pendulum which is inherently unstable due to its physics. Therefore, diagonal line features reflect the performance in dealing with small perturbations, the longer the diagonal line lengths are, the better the performance of balance control. In contrast, lower percentage determinism and shorter mean diagonal line length would reflect less predictable (i.e., more sensitive to small perturbations, lower balance performance) and more random CoP motion.

*Vertical line features*. Vertical line features reflect intermittent (laminar) behavior of CoP motion (Figures 1C,D). The percentage of recurrences that form vertical lines (%LAM) and the mean vertical line length (TT) measures intermittent behavior of CoP motion. Intermittent behavior reflects CoP motion that now and again exhibits changes in CoP dynamics from fluctuating to relatively stationary. For example, a vertical line occurs when a balance solution (state) revisits (Figures 1C,D, red dot CoP position example) a region in phase space, but then remains in that region for some time (Figures 1C,D, red line CoP motion example). A period of minimal change in balance states reflects balance that did not require substantial corrections during that time period. The length of these time periods, as measured by TT, reflects the presence of a point attractor, presumably a stable static state. Low laminarity and shorter mean vertical line lengths reflect balance control with fewer static states.

**Figure 2:**
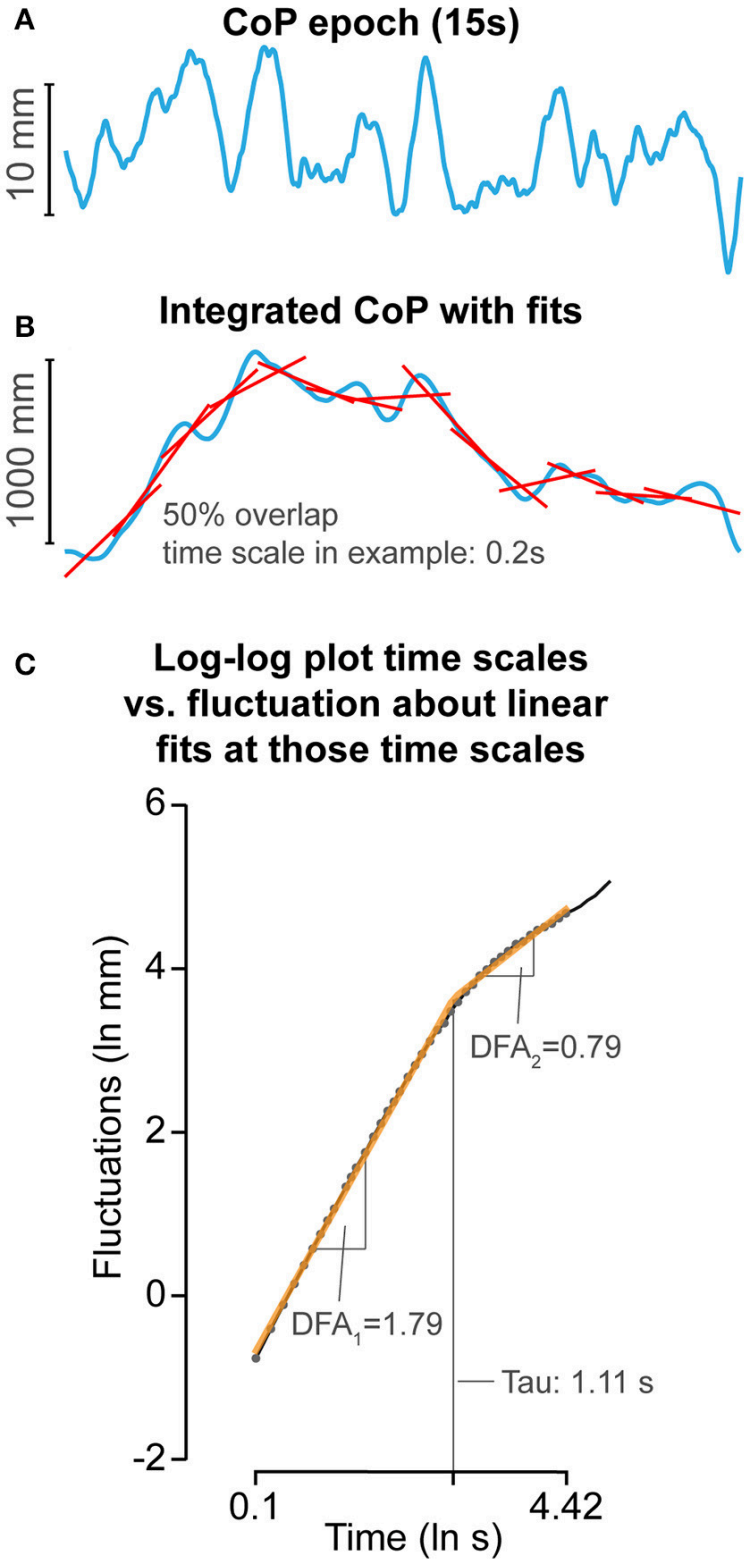
Detrended fluctuation analysis (DFA) methods. **(A)** Example of a 15-s CoP motion (see Figure 1A in blue). **(B)** CoP was integrated, then fluctuations of CoP around linear fits over windows ranging from 0.10 to 4.42 s were determined with 50% overlap. Example of 0.2 s is given. **(C)** Log-log plot of time windows vs. fluctuations. Two linear regions were fit by minimizing the squared errors between the combined linear fits and actual data. DFA_1_ and DFA_2_ reflect the general organization of fluctuations at shorter and longer time scales, respectively. DFA_tau_ reflects the time scale between DFA_1_ and DFA_2_.

*Detrended fluctuation analysis*. DFA measures the long-range dependence in signals, also referred to as “memory” (Peng et al., 1998). DFA measures the exponential relation between CoP fluctuations at different time windows (time scales) by measuring the slope of a linear region on the log-log plot of CoP fluctuations vs. time scales (Figure 2). The slope reveals the general organization of these fluctuations across a range of time scales. For example, a steeper slope of the exponential relation between CoP fluctuations at different time scales reflects CoP motion in which relative contribution of fluctuations at shorter time scales are less than fluctuations at longer time scales or vise versa. With this particular organization of fluctuations across the timescales, CoP motion appears to be smoother and tends to continue to move (persist) in the same direction (Mandelbrot, 1982), reflecting CoP motion that did not involve many direction changes. Figure 2 shows the technical details and settings of the used DFA method, and Table 1 provides definition and interpretations of DFA parameters used in the current study.

Briefly, the CoP signal was integrated over time to allow assessment of fluctuations at longer time scales (Delignières et al., 2010). The signal was then divided into smaller time windows with 50% overlap. In each time window, the linear trend was subtracted and the RMS fluctuations of the integrated CoP around the linear fits were determined. The window sizes ranged from 0.10 to 4.42 s. DFA has similarities with spectral analysis (Buldyrev et al., 1995), fluctuations within each window represent fluctuations at a frequency that can be captured within the time window of interest. Therefore, fluctuations within the 0.10–4.42 s windows represent fluctuations at frequencies ranging from 10 to 0.23 Hz. The underlying assumption is that CoP motion reflects a form of Brownian motion, and whether Brownian motion is persistent or anti-persistent is reflected by the slope of the fluctuations across multiple time scales (slope: anti-persistent <0.5–>0.5 persistent). However, although Brownian motion and non-stationary processes do not need to be integrated over time (Riley et al., 2012) as they are unbounded (i.e., Brownian motion is the integrated form of fractal Gaussian motion), it is beneficial to integrate CoP motion over time to allow assessment of fluctuations at larger time scales because CoP motion is bounded by the support surface area. However, because CoP motion is bounded and non-stationary, integration will consequently inflate the slope of the log-log plot of time-scales vs. fluctuations (Riley et al., 2012). Therefore, the slope will be interpreted like integrated Brownian motion. When the slope >1.5, a change in CoP movement is likely to be followed by a change in the same direction (CoP is persistent). If the slope alpha <1.5, a change in CoP motion is likely to be followed by a change in the opposite direction (CoP is anti-persistent). If alpha = 1.5, then a change in direction of CoP motion does not depend on previous directional changes, and relative contributions of fluctuations at different time scales are equal. Inspection of the log-log plot of time scale vs. fluctuations revealed a bilinear pattern (Figure 2). Hence, we calculated the slopes at shorter (DFA_1_) and longer (DFA_2_) time scales and the time point that marked the boundary between the two regions (DFA_tau_). The slope (DFA_2_) of the second region was in general smaller than 1.5, indicating that fluctuations at these time scales reflected CoP motion that tends to turn back toward the point it came from. Consequently, DFA_tau_ reflects the time scale at which persistent CoP motion changes into anti-persistent motion. Smooth COP dynamics, without large corrections, would be represented by a greater DFA_1_, DFA_2_, and DFA_tau_. A shorter DFA_tau_ and lower DFA_2_ values would reflect a more conservative balance strategy with early and strong CoP corrections.

The bi-linear pattern was determined as follows; two linear regions were fit on the log-log plot of time scales vs. fluctuations data by minimizing the squared errors between the combined linear fits and actual data. The region of the first linear fit (shorter time scales) was defined as DFA_1_ and the region of the second linear fit (longer time scales) was defined as DFA_2_. The time point separating these two linear regions was defined as DFA_tau_.

### Statistics

Matlab was used to perform the statistical analysis. The threshold for significance was set at *P* < 0.05.

#### Demographics

Differences between the demographics and PPA of the fallers and non-fallers were assessed using dependent *t*-tests and Chi^2^ for sex.

#### CoP motion at baseline

Differences between groups at baseline (15 s epoch before vibration) for each outcome variable (linear and non-linear) were assessed using one-way analysis of variance (ANOVA). *Post-hoc* analysis was performed as appropriate with Bonferonni correction for multiple comparisons.

#### CoP motion during and after vibration

Differences between fallers, non-fallers, and young for each of the non-linear and linear outcome measures were tested using a wavelet based linear mixed models (adapted from McKay et al., 2013). Wavelet based compression of the data reduces the number of significant *P*-values and therefore increases the statistical power (McKay et al., 2013). Briefly, data from the windowed analysis including two repetitions (2 × 32 data points) for each participant of the vibration epoch and the post vibration epochs (Figure 1A), were subjected to a level 1 wavelet transform using the Haar wavelet with periodic extension. For each wavelet coefficient, a linear mixed model was applied to assess differences between groups. Group and repetition were entered as fixed factors and participants were entered as random factors in the linear mixed model. Coefficients reflecting the differences between groups (young vs. non-fallers, young vs. fallers, and fallers vs. non-fallers) were assessed and corresponding *P*-values were stored. All *P*-values were then corrected for multiple comparisons using the Benjamini–Hochberg false discovery rate procedure (Benjamini and Hochberg, 1995). Wavelet coefficients representing significant group differences were then transformed back into the time domain.

## Results

### Falls incidence

Four elderly participants withdrew from the study (1 < 0.5 PPA, and 3 > 1.16 PPA), and no prospective falls data were available for these participants. Forty-two out of 102 elderly participants reported 1 or more falls. Of these participants, 2 reported 6 falls, 1 reported 5 falls, 3 reported 4 falls, 6 reported 3 falls, 9 reported 2 falls, and 21 participants reported 1 fall. Using the prospective falls data, the elderly were grouped into fallers (1 or more prospective falls) and non-fallers. Due to technical issues with data collection, data from 3 participants (1 faller and 2 non-fallers) were excluded from further analysis. Table 2 shows means (SD) of demographics and PPA values of all elderly participants included in the final analysis (*n* = 99). Age, height, weight, sex, and PPA values did not differ significantly between fallers and non-fallers (all, *P* > 0.09, Table 2).

**Table 2:** Participant demographics.

	**n**	**Height (m)**	**Weight (kg)**	**Age (years)**	**Gender**	**PPA**
Fallers	41	1.70 (0.08)	78 (17)	76 (5)	13 ♀, 28 ♂	0.86 (1.00)
Non-fallers	58	1.68 (0.10)	79 (15)	75 (6)	27 ♀, 31 ♂	0.52 (1.00)
*P*-value		0.26	0.79	0.51	0.14	0.09
Young	23	1.73 (0.5)	66.6 (11.3)	21.1 (1.5)	15 ♀, 9 ♂	

*Data are presented as mean ± standard deviation, probability (P) of independent 2-tailed t-tests (Chi^2^ for gender) between fallers and non-fallers are shown. ♀ represents female, and ♂ represents male*.

### CoP motion at baseline

Results of the one-way ANOVA are presented in Table 3. At baseline (before vibration), compared to fallers, young had lower SP (moved slower), lower %DET (were more predictable), had longer L_mean_ (less sensitive to small perturbations, i.e., better balance performance) and had higher %LAM with longer TT (were more intermittent, with longer static episodes). Regarding DFA analysis, compared to fallers, young exhibited a larger DFA_tau_ and DFA_2_ (a less conservative balance control strategy, lower anti-persistence: young>fallers) and had a larger DFA_1_ (smoother and more persistent: young>fallers). Compared to non-fallers, young also had lower SP (moved slower), longer L_mean_, longer TT, and were less anti-persistent (DFA_2_: young<non-fallers), but %DET, %LAM, DFA_1_, and DFA_tau_ were not significantly different between young and non-fallers.

**Table 3:** One-way analysis of variance between young (y), fallers (f) and non-fallers (nf) at baseline.

	**Oneway ANOVA**					***post-hoc***		
			**Mean (95% CI)**			***P*****-value**		
**Variable**	**F**	***P***	**y**	**f**	**nf**	**y-f**	**y-nf**	**f-nf**
SP (mm)	14.08	**0.000**	6.71 (3.33)	14.91 (13.02)	14.03 (14.01)	**0.000**	**0.000**	0.869
%DET	5.85	**0.004**	0.91 (0.09)	0.86 (0.15)	0.88 (0.11)	**0.003**	0.172	0.128
L_mean_	12.89	**0.000**	39.49 (16.01)	30.28 (12.02)	32.64 (14.03)	**0.000**	**0.000**	0.275
%LAM	7.05	**0.001**	0.95 (0.06)	0.88 (0.17)	0.91 (0.12)	**0.001**	0.065	0.142
TT	13.80	**0.000**	35.91 (17.57)	24.88 (14.05)	27.55 (16.68)	**0.000**	**0.000**	0.299
DFA_1_	4.05	**0.020**	1.81 (0.10)	1.74 (0.22)	1.77 (0.17)	**0.017**	0.290	0.280
DFA_2_	6.35	**0.002**	1.31 (0.40)	1.10 (0.46)	1.14 (0.47)	**0.002**	**0.011**	0.792
DFA_tau_	3.52	**0.033**	0.95 (0.83)	0.72 (0.55)	0.77 (0.65)	**0.033**	0.087	0.890

Significant differences are shown in bold font. Recurrence quantification analysis (RQA), diagonal line structures; percentage determinism (%DET), mean diagonal line length (L_mean_), vertical line structure; percentage laminarity (%LAM), mean vertical line length (trapping time, TT). Detrended fluctuation analysis (DFA), short term DFA (DFA_1_), longer term DFA (DFA_2_), and the time scale that separates DFA_1_ and DFA_2_; DFA_tau._

No differences between fallers and non-fallers were observed at baseline.

### CoP motion during and after vibration

**Figure 3:**
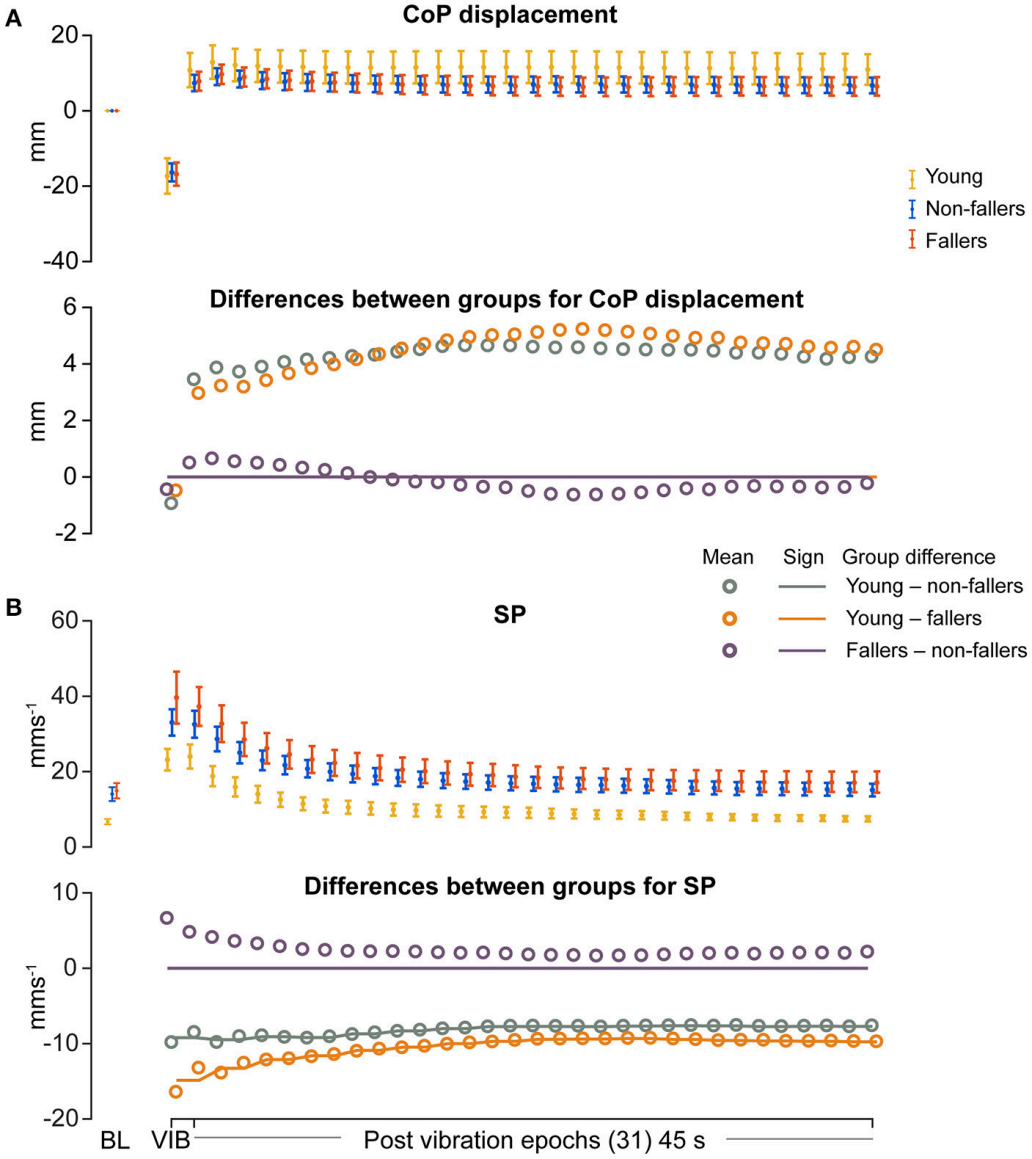
Results of linear measures during vibration (VIB) and post-vibration epochs. Top panel show mean for each group of **(A)** mean center of pressure (CoP) displacement and **(B)** sway path length (SP). Bottom panel in **(A,B)** show mean differences between groups (open dots), and the significant (sign) difference between groups (solid lines). Note that group differences are significant when the solid line matches the open dots. Group differences at baseline (BL) were assessed separately and are presented for visual reference. Error bars represent 95% confidence interval (1.96 × standard error of measurement).

**Figure 4:**
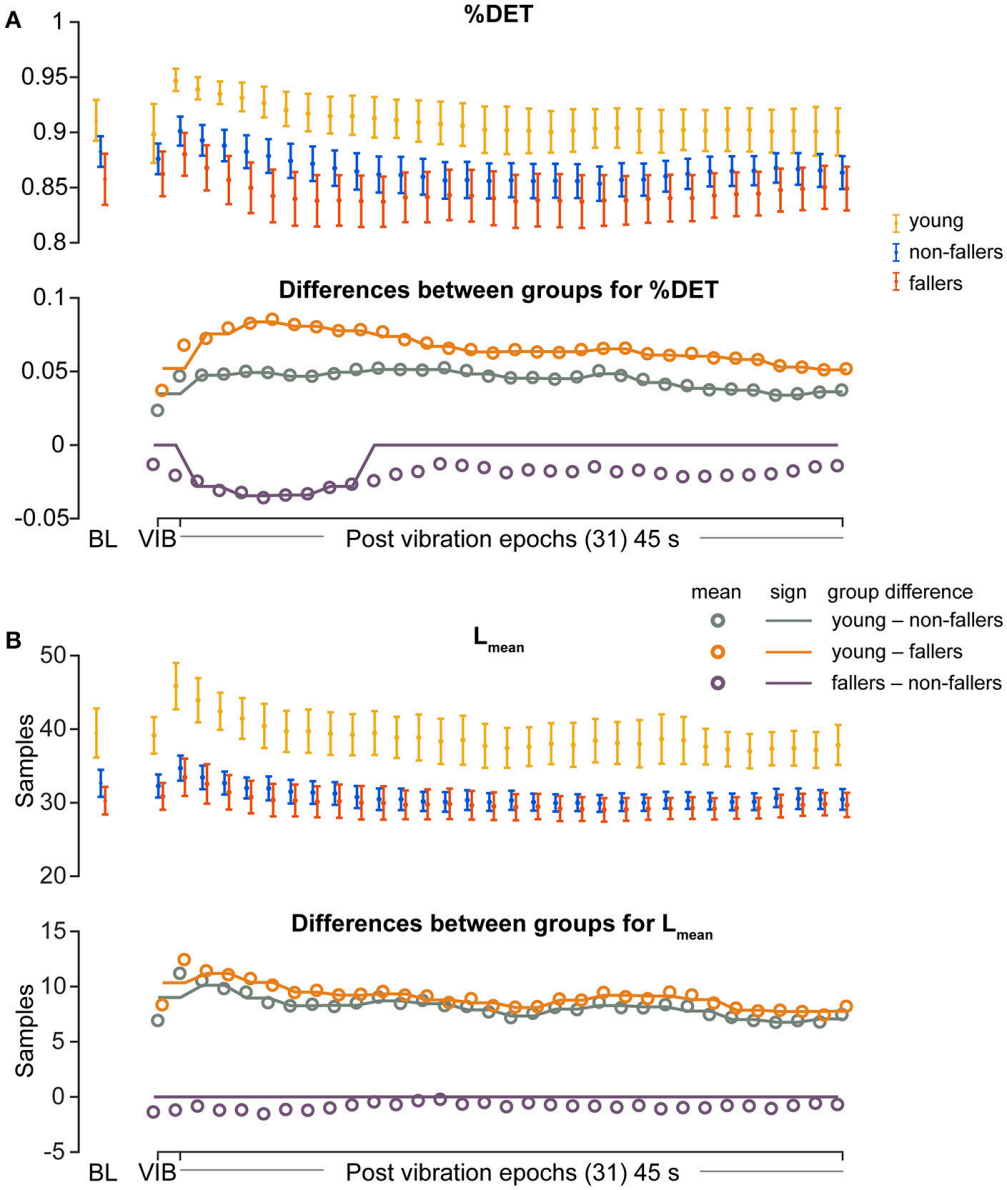
Results of recurrence quantification analysis (RQA) of diagonal line features during vibration (VIB) and post-vibration epochs. Top panels show mean for each group of **(A)**, mean percentage determinism (%DET) and **(B)** mean diagonal line lengths (L_mean_). Bottom panels in **(A,B)** show mean differences between groups (open dots), and the significant (sign) difference between groups (solid lines). Note that group differences are significant when the solid line matches the open dots. Group differences at baseline (BL) were assessed separately and are presented for visual reference. Error bars represent 95% confidence interval (1.96 × standard error of measurement).

**Figure 5:**
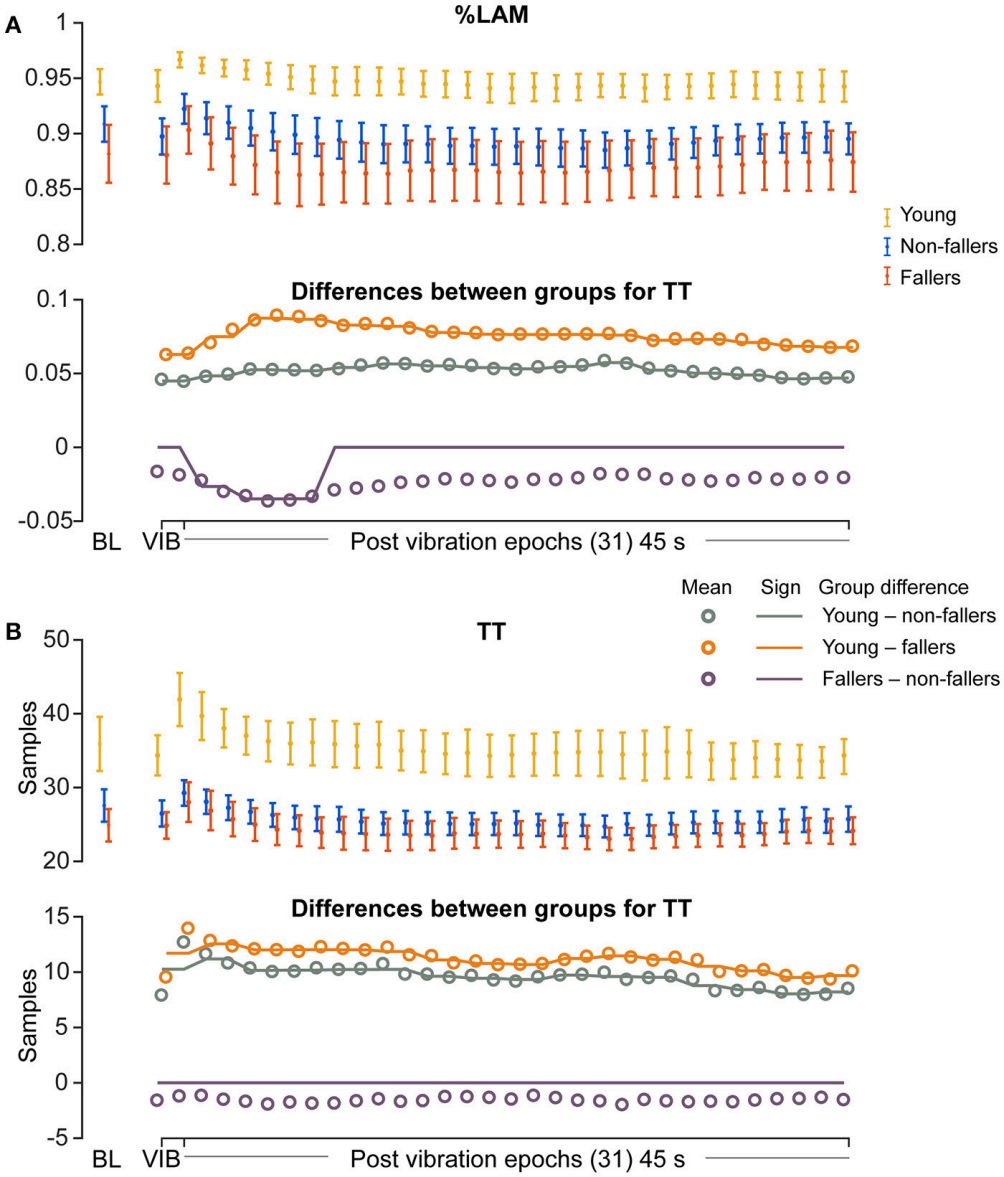
Results of recurrence quantification analysis (RQA) of vertical line features during vibration (VIB) and post-vibration epochs. Top panels show mean for each group of **(A)** mean percentage laminarity (%LAM) and **(B)** mean vertical line lengths (TT). Bottom panels in **(A,B)**, mean differences between groups (open dots), and the significant (sign) difference between groups (solid lines). Note that group differences are significant when the solid line matches the open dots. Group differences at baseline (BL) were assessed separately and are presented for visual reference. Error bars represent 95% confidence interval (1.96 × standard error of measurement).

**Figure 6:**
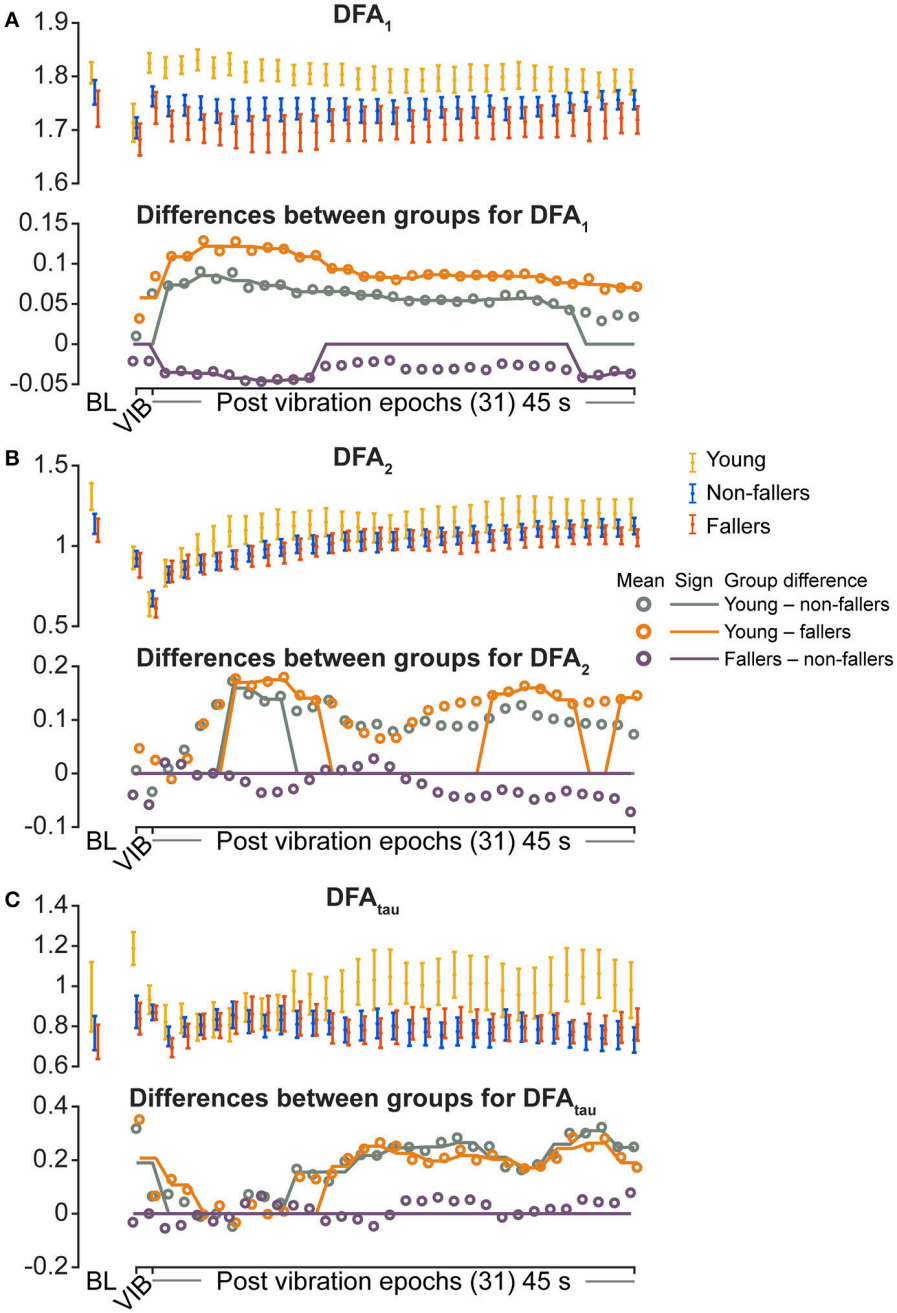
Results of detrended fluctuation analysis (DFA) during vibration (VIB) and post-vibration epochs. Top panels show mean for each group of **(A)** DFA_1_ (short term), **(B)** DFA_2_ (long term), and **(C)** DFA_tau_ (time scale that separates DFA_1_ and DFA_2_). Bottom panels in **(A–C)** show mean differences between groups (open dots), and the significant (sign) difference between groups (solid lines). Note that group differences are significant when the solid line matches the open dots. Group differences at baseline (BL) were assessed separately and are presented for visual reference. Error bars represent 95% confidence interval (1.96 × standard error of measurement).

Outcome data for CoP motion during and after vibration are shown in Figures 3–**6**, and summarized in Table 4. CoP mean displacement relative to baseline in response to calf vibration was not significantly different between the groups (Figure 3). In addition, CoP displacement after vibration was also not significantly different between the groups (Figure 3, Table 4).

**Table 4:** Overview of CoP variables that were different between young, fallers, and non-fallers.

		**Young vs. fallers**		**Young vs. non-fallers**		**Fallers vs. non-fallers**	
**Measure**	**Variable**	**VIB**	**Post VIB**	**VIB**	**Post VIB**	**VIB**	**Post VIB**
Linear	Displacement	NS	NS	NS	NS	NS	NS
	SP	Y>F	Y>F all epochs	Y>NF	Y>NF all epochs	NS	NS
RQA diagonal	%DET	Y>F	Y>F all epochs	Y>NF	Y>NF all epochs	NS	F<NF at epoch 2–9
	L_mean_	Y>F	Y>F all epochs	Y>NF	Y>NF all epochs	NS	NS
RQA vertical	%LAM	Y>F	Y>F all epochs	Y>NF	Y>NF all epochs	NS	F<NF at epoch 2–7
	TT	Y>F	Y>F all epochs	Y>NF	Y>NF all epochs	NS	NS
DFA	DFA short	NS	Y>F at epochs 2–31	NS	Y>NF at epochs 2–27	NS	F<NF at epochs 2–11, 28–31
	DFA long	NS	Y>F at epochs 6–11, 22–27, 30, 31	NS	Y>NF at epochs 6–9	NS	NS
	DFA tau	Y>F	Y>F at epochs 1, 2, 11–31	Y>NF	Y>NF at epochs 11–31	NS	NS

Recurrence quantification analysis (RQA), diagonal line structures; percentage determinism (%DET), mean diagonal line length (L_mean_), vertical line structure; percentage laminarity (%LAM), mean vertical line length (trapping time, TT). Detrended fluctuation analysis (DFA), short term DFA (DFA_1_), longer term DFA (DFA_2_) and the time scale that separates DFA_1_ and DFA_2_; DFA_tau._

For the young group, compared to fallers and non-fallers, across all epochs, %DET and L_mean_ (balance performance; young>fallers/non-fallers, Figure 4), %LAM and TT (intermittent control; young>fallers/non-fallers, Figure 5), and DFA_1_ (smoothness/persistence; young>fallers/non-fallers at most epochs, Figure 6) values were higher, and SP (young<fallers/non-fallers, Figure 3) was lower (Table 4). DFA_2_ was greater in the young than both fallers and non-fallers after removal of vibration at most epochs (Figure 6, Table 4). DFA_tau_ was higher in the young during vibration than both fallers and non-fallers, but, was similar for all groups directly after removal of vibration. DFA_tau_ values were higher in the young than both fallers and non-fallers after epoch 12 (Figure 6). This suggests that during vibration young were more persistent across more time-scales compared to both fallers and non-fallers (DFA_tau_: young>fallers/non-fallers).

During ankle vibration, fallers and non-fallers were not significantly different in any of the linear or non-linear outcome variables (Figures 3–6). However, after removal of ankle vibration, CoP motion of the fallers had lower %DET (less predictable/more random; fallers<non-fallers, Figure 4), lower %LAM (less intermittent; fallers<non-fallers, Figure 5), and lower DFA_1_ (less smooth/less persistent; Figure 6) values than those in non-fallers. See Table 4 for overview of the findings.

## Discussion

This study assessed the effect of addition and removal of calf vibration on balance control (in the absence of vision) in elderly people who did or did not prospectively report falls and a group of young individuals. Mean CoP displacement during and after vibration did not differ between groups, suggesting that peripheral functioning of calf proprioception and weighting assigned by the CNS to calf proprioception were similar for the young and both groups of older individuals. Overall, compared to the elderly, the CoP motion of young was more predictable and less sensitive to small perturbations. Notably, non-linear aspects of CoP motion of fallers differed from that of non-fallers after removal of vibration, a period in which dynamic re-weighting is required (Teasdale and Simoneau, 2001). During this period fallers exhibited more random CoP motion.

### Interpretation of non-linear measures of CoP motion

Our conclusions are based on interpretation of the non-linear measures of CoP motion, yet alternative interpretations of some RQA measures requires consideration (Negahban et al., 2010; Ramdani et al., 2013; Bernard et al., 2015). In contrast to our interpretation that a more deterministic structure infers more regular and stable balance control, this regularity has also been interpreted to reflect less behavioral flexibility and less complexity in CoP motion. That alternative interpretation was motivated by observations of increased regularity of CoP motion in Parkinson's disease (Schmit et al., 2006), retrospectively identified fallers (Ramdani et al., 2013), and in individuals with anterior cruciate ligament deficiency (Negahban et al., 2010). Although these interpretations might be reasonable in some cases (e.g., Parkinson's Disease), it might not apply to our observations.

Our interpretations and of others (Riley et al., 1999; Riley and Clark, 2003; Haddad et al., 2008; Cluff et al., 2009; Mazaheri et al., 2010) are based on the construct validity of RQA, methodological issues, and the nature and difficulty of our balance task. The deterministic structure is related to diagonal line feature in the recurrence plot. Both %DET and L_mean_ reflect CoP motions that run parallel in phase space because different CoP sections are spatially and temporally similar in shape (Figures 1C,D). This similarity reflects predictability, regularity and local stability of CoP motion. A lower %DET and shorter L_mean_ does not necessarily reflect more complex structure because random signals and noise also exhibit low %DET, yet those signals do not have a complex structure. Thus, although “predictability” can be determined, care is required to interpret “flexibility” and “complexity” from RQA outcome variables.

Interpretation of RQA can be aided by concurrent assessment of other non-linear features using additional measures such as DFA, as we have done. Our observation that young people have higher long-range correlations than elderly concurs with other observations of similar differences between young and old (Norris et al., 2005; Amoud et al., 2007; Duarte and Sternad, 2008; Kim et al., 2008). Observed CoP fluctuations reflect both the perturbations [internal (self-generated) and external] and the postural responses to perturbations. The combination of these perturbations is resolved by the postural system. When the postural system appropriately responds to perturbations, then successive CoP positions relate to previous positions and correlation is strong (Bruijn et al., 2013). Successful balance corrections to perturbations would not likely result in additional changes of CoP direction, but instead induce smooth CoP motion that tends to persist in the same direction. Alternatively, more changes in direction would increase the relative contribution of CoP fluctuations at shorter time-scales (DFA_1_) and reduce the strength of long-range correlations. CoP motion with fewer directional changes/smoother appearance would more likely be more deterministic than CoP with more directional changes. Thus, DFA outcomes aid interpretation of whether the deterministic structure of signals arise from regular signals containing fluctuations at a single or limited number of time scales (such as a sine wave), or from regular signals with long-range correlations across multiple time scales. Taken together, concurrent observations of RQA and DFA strengthen our interpretation that high %DET and long L_mean_ observed in CoP motion of the young compared to the elderly is not related to reduced complexity as underlying CoP fluctuations were evident at a range of time scales.

Some RQA settings require consideration as they could impact findings. Without an appropriate corridor (Theiler window, shown in Figure 1C) along the line of identity (i.e., self-recurrences of CoP states), diagonal line measures could be overestimated by the inclusion of recurrences that are temporally close (Marwan, 2011). This is important as the threshold, below which a recurrence is defined, depends on the phase space diameter (i.e., the amplitude of CoP movements) or depends on the fixed amount of recurrences in the recurrence plot. In both cases, higher amplitude CoP motion, resulting in a larger phase space diameter, would require a higher recurrence threshold. This would increase the neighborhood to find recurrences. Greater amplitude of CoP motion is usually observed in elderly (Hageman et al., 1995; Gill et al., 2001; Abrahamová and Hlavačka, 2008). In these cases, RQA would be biased to observe longer diagonal lines and higher determinism if an appropriate Theiler window was not used (Marwan, 2011) allowing inclusion of temporally close neighbors. We used a 1-s Theiler window to minimize this bias. Use of a Theiler window was not reported in previous studies (Seigle et al., 2009; Ramdani et al., 2013; Bernard et al., 2015), the max diagonal lines reported in some studies (Riley and Clark, 2003; Schmit et al., 2006) generally approach the length of the delay embedded signals, which implies that a Theiler window was not applied and findings may have been biased.

The difficulty of the task used to assess balance control requires consideration. In the current study, participants maintained balance while somatosensory information from the calf muscle was suddenly altered by addition and removal of vibration in the absence of vision. Although aware of the experimental conditions, participants were in an unfamiliar balance situation and none had previously experienced muscle vibration. When balance is challenged, more predictable balance control could be beneficial. Increased regularity and smoothness of CoP motion could be interpreted as an appropriate adaptation to a more challenging balance task (Riley et al., 1999).

### Effect of vibration on postural control

The postural vertical is modified when sensory input is increased by muscle vibration (Eklund, 1972). The impact of vibration depends on both peripheral functioning of the muscle spindles and the weighting of this sensory input. Our results showed, on average, that linear measures of the CoP displacement caused by addition and removal of calf vibration did not differ between the young group and both older groups. This suggests the peripheral functioning of the calf proprioceptors and the weight assigned to the sensory information were not different between the groups. Although, Brumagne et al. (2004) also found similar CoP displacement for older and healthy young individuals, some authors (Pyykkö et al., 1990; Quoniam et al., 1995) reported less CoP response to calf vibration with increased age. Pyykkö et al. (1990) and Quoniam et al. (1995) used older individuals from a non-community dwelling setting which might explain the difference in outcome. Further, Quoniam et al. (1995) used 3 s vibration (15 s used here), which might be too short (Capicíková et al., 2006) to affect their elderly group's CoP.

Although addition of vibration did not differently perturb balance in fallers and non-fallers, removal of the vibratory stimulus revealed differences. This implies that dynamical integration of sensory information is more challenging when sensory input is reduced than when it is augmented. This appears to concur with observations that, in some contexts, addition of a subthreshold stochastic stimulus (e.g., vibration shoe soles) can improve balance (Niemi et al., 2002; Priplata et al., 2003). Although we did not observe improvement of balance in response to sudden unfamiliar addition of a supra-threshold vibratory stimulus to the muscle, our data did show that both fallers and non-fallers responded equally well to this perturbation (across our suite of measures). Spindle activity related to changes in calf muscle length is likely to be masked during vibration (Roll et al., 1989) indicative of reweighting of the calf proprioception by the CNS away from the additional inaccurate component provided by the vibration. Although inaccurate information is provided, the additional input was integrated by the CNS for balance control evident by the backwards CoP shift in all groups. Cessation of vibration ceases the vibration related discharges immediately (Roll et al., 1989). Because the proprioceptive information was down-weighted during vibration, sudden reduction of proprioceptive information may be more difficult to accommodate to than adding proprioceptive information using vibration. The period after removal of vibration would require fast reweighting to use available somatosensory information (van der Kooij and Peterka, 2011) and the proprioceptive information from the calf might contain greater noise (Rogers et al., 1985) and less useful somatosensory information than during vibration. Further, analysis using the sliding window after removal of vibration may have reduced the variance leading to a greater probability to observe significant differences between groups than the analysis of a single epoch during vibration.

SP is known to increase, together with L_mean_ and %DET, in balance tasks with greater sensory challenge (e.g., eye closure, compliant surfaces; Riley et al., 1999; Riley and Clark, 2003). Increased sway would generate more proprioceptive information and potentially compensate for reduction/removal of sensory information from other sources (Carpenter et al., 2001, 2010). This “self-generated” proprioceptive information is dynamically integrated to guide balance control leading to more deterministic CoP motion and has been referred to as “perceptually guided control” (Riley et al., 1999). Although our elderly group had longer SP than the young group, this was not associated with more deterministic CoP motion. A similar observation was made by Seigle et al. (2009); removal of vision in quiet stance led to greater SP but less %DET in elderly than younger individuals. This highlights a potential compromise in perceptually guided control in the elderly; particularly for fallers after removal of vibration.

Lower DFA_2_ and DFA_tau_ in all groups after vibration removal suggest that fluctuations at longer timescales were relatively small (~>0.8 s from DFA_tau_). Proprioceptive information that establishes sense of upright functions at lower frequencies (Diener et al., 1986; Diener and Dichgans, 1988), and reduced fluctuation at longer time scales could reflect that the vertical upright of the participants was affected after removal of calf vibration. The observation of greater DFA_2_ and DFA_tau_ for young than older participants after ~15 epochs (window starting 15 s after cessation of vibration) suggests that young more successfully established an upright subjective vertical. As we assessed fluctuation at a maximum time scale of 4.42 s our data might partly reflect recalibration of upright sense by dynamic reweighting of sensory information that establishes upright sense (i.e., proprioceptive information from the calf and vestibular input).

Poorer balance control after removal of vibration in fallers might be related to an inferior capacity to dynamically reweight the sources of somatosensory information to minimize the perturbation effects of vibration removal. The ability to flexibly explore somatosensory information is affected by increased age (Teasdale et al., 1991; Hay et al., 1996; Doumas and Krampe, 2010; Eikema et al., 2013, 2014). Reduced functioning of somatosensory systems other than the calf muscle spindles might also affect balance steadiness. Physiological aging affects all somatosensory systems (Sturnieks et al., 2008), particularly the vestibular system (Sloane et al., 1989; Strupp et al., 1999) as evidenced by greater dependence of elderly individuals on visual information to establish vertical upright (Sundermier et al., 1996; Simoneau et al., 1999), and inferior capacity to align themselves with the vertical after being tilted (Menant et al., 2012). Because sensory information from the calf was unreliable and vision was unavailable in the present study, reliance on vestibular information would have been increased. Compromised function of the vestibular system would also render the somatosensory information less useful, as the internal upright reference frame against which somatosensory information is compared (Horak et al., 1989) is less accurate. Accurate sense of vertical in combination with optimal balance control would be expected to produce CoP motion that exhibits periods of minimal CoP movements and high %LAM as observed in the young group. It follows that lower %LAM observed in the older group, and lower %LAM observed post vibration in fallers than non-fallers might reflect a compromise of this combination. Confirmation that %LAM relates to inaccurate upright sense, poorer balance control, or both cannot be derived from the present data and requires further investigation.

### Fractal nature of CoP motion

#### Balance between order and disorder

Delignières et al. (2011) hypothesized that systems exhibiting long range correlations are flexible and adaptable and are more robust. Long range correlations are argued to stem from the collective behavior of multiple components within the system (Peng et al., 1995a) that partially overlap in functionality generating a multi-scaled and hierarchical structure (Delignières and Marmelat, 2013). The lack of a characteristic scale would help prevent a single steady state (excessive mode locking) restricting the functional responsiveness of the system (Peng et al., 1998; Goldberger et al., 2002). The relation of fluctuations at the different scales can be assessed with DFA, however, why the sum of these various contributions is scaling is unclear. Systems with long range correlations fall between systems with too strict control exhibiting excessive order, and systems with no control exhibiting disorder (Peng et al., 1998; Delignières and Marmelat, 2013). The elderly's CoP motion with weaker long-range correlations in combination with lower %DET than young, could suggest that balance in elderly was less controlled, and more stochastic. This observation was exacerbated in fallers during a period after cessation of calf vibration compared to non-fallers and suggests that fallers' balance was more disorderly (i.e., less controlled) than non-fallers during this time period.

#### Underlying balance control mechanisms

A wide variety of physiological processes exhibit complex fluctuations that obey scaling laws describing their fractal nature (Goldberger et al., 2002). Most of these physiological processes are affected by pathology and/or physiological aging (Goldberger et al., 2002). Although the origin of these fluctuations is largely unknown, the organization of these fluctuations is not random. Instead, fluctuations exhibit correlations over a wide range of time-scales and can exhibit different multifractal complexity levels (Ivanov et al., 2009) providing information on the underlying control mechanisms (Ashkenazy et al., 2002; Goldberger et al., 2002; Hu et al., 2004; Ivanov et al., 2009). For instance, the heart beat is regulated by the autonomic nervous system and changes in its control affect the variability of the time between beats (Goldberger et al., 2002). Similarly, the complex central control of gait results in complex variability of the time between consecutive gait cycles (Hausdorff et al., 1995). For example, the correlation of stride time variability and fractal complexity reduces with maturation (Hausdorff et al., 1999; Ashkenazy et al., 2002) and is affected by aging and disease (Hausdorff et al., 1997). These alterations are argued to stem from changes of the CNS possibly reflecting stronger or reduced connections between different parts that control walking (Ashkenazy et al., 2002).

Some potential parallels can be drawn between the observation of changes in stride time variability with aging (Hausdorff et al., 1997). Aging is related to a narrowing of the physiological functional range (Rosenberg, 1989; Shaffer and Harrison, 2007) related with a decline in morphology and physiological functioning of the sensory system (Shaffer and Harrison, 2007), central processing of sensory information (Goble et al., 2011), and muscular system (Rosenberg, 1989). The aging process results in structural and functional changes, which limit the responsiveness and flexibility of the balance control system. In line with Ashkenazy et al. (2002) and Hausdorff et al. (1997), DFA values in the present study were lower in elderly than young and were lower in fallers than non-fallers after removal of the vibratory stimulus. These observations suggest a simpler structure and function of the underlying control system in elderly individuals, which was amplified after vibration in fallers compared to non-fallers. Future investigation is required to identify the non-linear properties that underlie the correlations in CoP motion fluctuations as measured by DFA. Preferably without external alteration of sensory information and with longer signal durations.

Although the underlying intrinsic control mechanism of a physiological process can be assessed by its fractal structure (Goldberger et al., 2002), DFA analysis of CoP motion assesses balance control strategies and therefore indirectly reflects the intrinsic structure of the underlying control system. Other factors that underpin CoP motion fluctuations could explain DFA observations. Underlying biomechanics of upright stance are reflected in CoP motion. Upright balance is thought to be intermittently controlled (Loram and Lakie, 2002; Vieira et al., 2012). Postural sway is probably continuously monitored but controlled by intermittent burst-like actions of postural muscles (Vieira et al., 2012). In between postural control actions, low amplitude CoP motion reflects the deterministic motion similar to the motion of an inverted pendulum (Asai et al., 2009). Intermittent control of upright balance could potentially explain the different scaling properties of CoP motion. In this view, DFA_1_ reflects the smooth persistent motion, consistent with that of an inverted pendulum, and DFA_2_ reflects the anti-persistent motion, consistent with intermittent postural corrections to limit center-of-mass within base of support. Elderly exhibited a more conservative balance strategy with less emphasis on persistence, in line with lower %DET, shorter L_mean_ and DFA_1_ in elderly than young, and more emphasis on anti-persistence in line with lower DFA_2_ during a period (~6–24 s) after vibration. Altered balance strategy observed in elderly individuals might reflect age-related changes in the intrinsic structure of the underlying control system.

## Conclusion

The present results show that non-linear measures of CoP motion, in response to a perturbation that challenges reweighting of integration of sensory input, reveal differences in the quality of balance control between young and old individuals and between older individuals who do and do not go on to fall. Consideration of the interpretation of non-linear measures provides new insights into the possible mechanisms underlying balance dysfunction and risk for falling in older individuals.

## Ethics statement

All subjects gave written informed consent in accordance with the Declaration of Helsinki. The protocol was approved by the University Human Research Ethics Committee of Queensland University of Technology and by the Medical Research Ethics Committee of The University of Queensland.

## Author contributions

WvdH: Study design, acquisition of data, data analysis, and interpretation, and drafted the manuscript; GK, JvD, and PH: Study design, data interpretation, and manuscript revision. All authors approved final version of manuscript.

### Conflict of interest statement

The authors declare that the research was conducted in the absence of any commercial or financial relationships that could be construed as a potential conflict of interest.
